# Correction: On the relation between transversal and longitudinal scaling in cities

**DOI:** 10.1371/journal.pone.0240758

**Published:** 2020-10-08

**Authors:** Fabiano L. Ribeiro, Joao Meirelles, Vinicius M. Netto, Camilo Rodrigues Neto, Andrea Baronchelli

The ORCID iDs are missing for the first and fifth author. Please see the authors’ respective ORCID iDs here:

- Author Fabiano L Ribeiro’s ORCID iD is: 0000-0002-2719-6061 (https://orcid.org/0000-0002-2719-6061).
- Author Andrea Baronchelli’s ORCID iD is: 0000-0002-0255-0829. (https://orcid.org/0000-0002-0255-0829).
