# Supplementary material for: On the relation between transversal and longitudinal scaling in cities
Source: PLoS One. 2020 May 19;15(5):e0233003. doi: 10.1371/journal.pone.0233003 (PMC7236989; doi:10.1371/journal.pone.0233003)
Supplement: Attachment — Submitted filename: to_referees___plos_one.pdf [file pone.0233003.s003.pdf]

February 27, 2020

Response to the Editor

We thank you warmly for the careful consideration of our manuscript. We have addressed all of the Reviewers' concerns as detailed below, and we hope that you may now find the paper suitable for publication.

1. *it is not fully clear whether the definition of the urban areas used in your analyses is a purely administrative or functional one, or a mix of the two.*

2. *results would hold only when cities actually grow. Would you be able to find the same results were shrinking cities also taken into account?*

3. *it would also be beneficial to better and more clearly position the paper within a Urban Economics perspective. The work of Henderson, Gabaix, and the theoretical models à la Fujita, Krugman, and Mori offer useful bridges in this respect.*

   Answer: Please, see our response to Reviewer 1.

4. *Please ensure that your manuscript meets PLOS ONE's style requirements, including those for file naming.*

   Answer: The new version of the manuscript reflects the style requirement (including file naming).

5. *In your Data Availability statement, you have not specified where the minimal data set underlying the results described in your manuscript can be found. Upon re-submitting your revised manuscript, please upload your study's minimal underlying data set as either Supporting Information files or to a stable, public repository and include the relevant URLs, DOIs, or accession numbers within your revised cover letter.*

   Answer: We have uploaded a spreadsheet (.ods file) to the Plos One submission web page containing all the data used in our work (please see supplementary material in the new manuscript version). It contains the data that we mined from sources IBGE and SNIS. It includes information for all Brazilian cities and their respective population size, GDP (from 1999 to 2014) and water network length (from 1995 to 2015).

6. *Thank you for stating the following in the Financial Disclosure section: "This work was supported by Swiss Mobiliar (Joao Meirelles), École polytechnique fédérale de Lausanne (Joao Meirelles), Conselho Nacional de*

Desenvolvimento Científico e Tecnológico (Fabiano Lemes Ribeiro, process number: 405921/2016-0), Coordenação de Aperfeiçoamento de Pessoal de Nível Superior (Fabiano Lemes Ribeiro, process number: 88881.119533/2016-01). The funders had no role in study design, data collection and analysis, decision to publish, or preparation of the manuscript." We note that you received funding from a commercial source: Swiss Mobiliar

Answer: We clarified this issue in the new manuscript version.

Response to Reviewer 1

1. *The paper looks at scaling behavior for 5507 municipalities - individual municipalities within cities are not whole urban units, and therefore the geographic definitions should be argued for and justified. (...) Is a municipality the same as a neighbourhood within a city, or an entire city? Explain upfront.*

   Answer: We thank the Reviewer for this critical comment. In fact, the word 'municipality' was used in an imprecise way in the first version of the manuscript. It was corrected in this new version. All presented analysis was done using 5507 'set of municipalities'.

   The raw data available (see spreadsheet attached) refers to every Brazilian municipality (that is, every single administrative unit). As we explain in the new version of the paper, a municipality is defined according to political and administrative matters operating over a territory that includes both urban and rural areas. So we preferred to use in our analysis 'aggregate sets of municipalities'. Each set represents all municipalities that are sufficient close to one each other in a way to form a single, continuous spacial urban unit: a single urban formation. Each set aggregates the information of all municipalities that belong to it. By far, most of these sets are formed by a single municipality, but some of the biggest Brazilian urban agglomerations are formed by more than one municipality, aggregated in metropolitan conurbations. This definition has led us from 5570 administrative municipalities to 5507 urban agglomerations as 'aggregate sets of municipalities', which we referred to simply as 'cities'.

   It is important to say that, according to our analysis, the qualitative results do not change if we use the 5570 municipalities or if we use the 5507 set of municipalities.

   Please see the blue text in section 'Materials and methods' of the new version.

2. *In author summary: the authors write: "while others like urban infrastructure would grow at a slower rate". this should be rewritten as "while others like urban infrastructure would grow but at less than proportional rates" (making the sublinear pattern clear). Similarly, "socioeconomic output would grow at a higher pace" should be something like "socioeconomic output would grow at a more than proportional rate".*

   Answer: Thank for these suggestions. We corrected these terms in the new version of the manuscript.

3. *The authors say (line 28) "The universality proposition has been challenged [26–28], but most evidence seem to confirm the generality, while exceptions are normally explained by local particularities [10, 18, 29, 30]". This is too strong and early to claim. For example, several research papers old and new show in fact that the population variable alone is insignificant in regressions when industrial or occupational organizations are considered, or statistical models other than the scaling model equally well explain the data. This statement thus needs to be softened - saying that this is as yet an open question, both domain wise and methodologically and theoretically:*

   Scaling and Hierarchy in Urban Economies,

   `https://arxiv.org/abs/1102.4101`

   Evidence for localization and urbanization economies in urban scaling,

   `https://arxiv.org/abs/1910.07166`

   Leitao, J. C., Miotto, J. M., Gerlach, M. and Altmann, E. G. 2016. Is this Scaling non-linear? Royal Society Open Science 3: 150649.

   Answer: Thanks once again for this suggestion, which was properly incorporated to the new version of the manuscript (see blue text in the Introduction section of the new version).

4. *Again, this is too naive: (line 33) "the total amount of social interactions between its citizens would guide, to a great extent, the city towards the observed scaling behavior". Economic and social organizations could be very different given the same group sizes or interaction densities. This claim seems to suggest that a 100 school teachers interacting is the same as a 100 finance and bankers interacting, and it simply does not matter what is being interacted upon!*

   Answer: The reviewer is right to say that the system as a whole responds different dependent on the kind of agents that are interacting. However, the data represent the average of all that is happening in the cities. The conclusion and claims are based on such average behavior across agents. Of course, it would be very interesting to isolate the contribution of different agents within the system (e.g. school teachers, bankers) to the city's overall output. But such an approach would need data at a very detailed microscopic level, which is probably unavailable or non-existent in the Brazilian context, other than data related to firms and economic industries, which lie outside our substantive and methodological scope.

5. *(line 34) "This proposition is unprecedented in urban science and the identification and validation of such universal dynamics could help urban policymakers to identify opportunities and improve the life quality of dwellers." The above is too vague and no claims on policy should be made without a deep and direct connection to how such (still dubious, open or unconfirmed) findings can actually help policy.*

   Answer: We agree with this observation, and attempted to clarify such connections in the new manuscript version. However, we must highlight

that a great number of recent works have suggested ways to use, or to get insights from, scaling laws to use in urban policy. In the new version we included a non-exhaustive example list (please see the text in blue in the Introduction).

6. *Overall, there is a crucial question that is missing here, and at least a qualitative discussion should be added to address this issue: the scaling equations as they currently stand, show only growth - which is fine as an approximation as far as transversal systems of cities are concerned. However, when it comes to individual cities, they can also "die" - in the sense that there is a process of growth, but there is also a process of decline and death. In such a case, do the authors hypothesise that declining populations and their corresponding urban variables would fit on the same law? Detroit? Etc.*

   Answer: We appreciate this observation. Unfortunately, the data does not allow us to reach any definitive position about the relationship of scaling laws and decreasing cities, since more than 70% of the Brazilian cities that are decreasing have less than 10,000 inhabitants. Also, very small cities do not allow good statistics for scaling. In addition, from a total of 677 decreasing Brazilian cities, only 3 have a population greater than 100,000 inhabitants. Regardless of this fact, this is a great question that deserves to be explored in other databases. A brief discussion about this was incorporated in section 'Theoretical Approach'.

Section: "Materials and Methods"

7. *Repeat but very important issue: for following point: (line 79) "The data presented here refer to 5507 Brazilian municipalities, with contiguous dense surrounding areas aggregated in single spatial units from the totality of 5570 Brazilian administrative divisions". Please provide justification that these can be considered as individual cities (why? - daily commute lengths are contained / population numbers / densities... what is the basis for defining each as an individual city, and how is this a reliable basis). The results in the entire paper rest on this crucial point.*

   Answer: See answer for the question 1.

8. *Figure 1: beta as 1.04 by the maximum likelihood method, which maximum likelihood method? Provide reference.*

   Answer: In the new manuscript version, we introduced details about how we used the maximum likelihood method to find the parameter β, along with some reference which corroborates the use of such method. Please see the blue text in section 'Results and Discussion'.

9. *1.04 is only mildly superlinear, so strong claims on super linearity will not hold.*

   Answer:

   We agree that 1.04 is only mildly superlinear and we tried to avoid 'strong' claims. However, we do believe that the evidence we provide supports the

statement about superlinearity. In particular,

1) GDP superlinearity has been shown several times in the literature, e.g. other works about Brazilian cities [1, 2], US cities [3, 4, 5], and european cities [6, 7];

2) We found a superlinear behaviour for all the analysed years (from 1998 to 2014);

3) In a previous work [1], it is shown that when it is considered only sufficiently dense cities, the GDP scaling exponent of Brazilian cities increases to 1.15.

Taken together, we believe that these three points support the thesis of a superlinear behaviour of GDP in the analyzed context.

Section: "Results"

10. *The authors should present some hypotheses and discussions on why they think that GDP (which is an economic variable) and the water supply network, which is a physical variable, should show differing relationships when compared with the transversal. They make the beginnings of this in considering city growth rates - but the main question is why does an economic variable behave different to a physical infrastructure variable?*

    Answer: The most plausible theory to explain it is the one proposed by Bettencourt et al. in 2007 [8, 3] (see also [9]), that socioeconomic variables, as GDP, is directly proportional to the number of human interactions in a city, which growths super-linearly with the population size. On the other hand, infrastructure variables present scaling economy with the population size, in the sense that bigger cities need less infrastructure per capita. This results in a sublinear behaviour between infrastructure and population size. This urban scaling economy is analogous (but only qualitatively) to what happens in biological systems: larger animals are more economical energetically since they spend less energy per unit of mass.

    It was included in the new manuscript version. Please see the blue text in the Introduction and Results sections.

Section: "Theoretical section"

11. *The formal part presented in the theoretical section is clear and nicely laid out. However, what I was not able to understand is - there are also old and established economic theories that discuss the growth of cities (for example, the entire body of work by Henderson, Gabaix, Krugman, Thisse, Fujita, Ogawa....) - how does the current naive physics of vector fields (which is not very physically motivated, but is a very 'data' based perspective) relate to the previous work done in detail on these questions? At present, the work feels overall like a naive exploration of data.*

    Answer: The reviewer has brought a critical issue, which is to highlight connections with a different field focused precisely on explaining how such scaling effects (as 'increasing returns') come into being. The new version

of the manuscript attempts to solve that issue (please see the Introduction). The statistical findings in the emerging field of scaling laws in urban science confirm earlier findings in spatial economics, namely in the field of agglomeration economies. We summarized some of the key findings, including works from the New Economic Geography school, works on Marshall-Arrow-Romer scale externalities related to size, density and specialization, and references on externalities based on spill-overs across industries.

Concerning the vectorial approach, we see it as a technical means to separate the effects acting in an urban metric in two parts: one that is pure scaling (internal factors); and another that takes into account external factors, for instance, the results of public policies. An example of the applicability of this approach was presented in the "The external Vector" section where we, along with Pulmain et al.'s theory, identified a possible example of infrastructure technology diffusion from large to small cities in the Brazilian context.

Response to Reviewer 2

1. *To include a discussion about the following paper: - The Interpretation of Urban Scaling Analysis in Time*

   `https://papers.ssrn.com/sol3/papers.cfm?abstract_id=3459540`)

   Answer: We thank the reviewer for informing us about such reference. There is a theoretical result in such reference (equation 17c) that is quite similar to the result that we have found (see Eq. 10 of our manuscript). This result was obtained independently. Once we learned about this work, we took great care to cite it properly. Please see blue text in the Introduction and Theoretical Approach sections.

2. *The sort of similar study (though with a different perspective) also looked at the evolution of urban scaling for several urban indicators for Brazilian data. - Scale-Adjusted Metrics for Predicting the Evolution of Urban Indicators and Quantifying the Performance of Cities*

   `https://journals.plos.org/plosone/article?id=10.1371/journal.pone.0134862`

   The authors could include a comment about this paper and explain the possible relation of its findings with your results.

   Answer: Thank very much for the suggestion. The reference was properly commented and cited in the new version. See the blue text in the Introduction section.

3. *There are some typos that should be fixed. For instance, page 7, "quantity Whose" or page 9, "the external Vector".*

   Answer: Thanks. This was corrected in the new version.

References

[1] J. Meirelles, C. R. Neto, F. F. Ferreira, F. L. Ribeiro, and C. R. Binder, "Evolution of urban scaling: evidence from Brazil," *PLoS One*, vol. 10, pp. 1–15, 2018.

[2] C. A. Ignazzi, "Scaling laws, economic growth, education and crime: Evidence from Brazil," *Espace géographique*, vol. 43, no. 4, p. 324, 2014.

[3] L. M. A. Bettencourt, "The origins of scaling in cities.," *Science*, vol. 340, no. 6139, pp. 1438–41, 2013.

[4] L. Bettencourt and G. West, "A unified theory of urban living.," *Nature*, vol. 467, pp. 912–3, oct 2010.

[5] C. R. Shalizi, "Scaling and Hierarchy in Urban Economies," vol. I, no. 1, pp. 1–15, 2011.

[6] L. M. A. Bettencourt and J. Lobo, "Urban Scaling in Europe," *J. R. Soc. Interface*, vol. 13, no. 20160005, 2016.

[7] E. Strano and V. Sood, "Rich and poor cities in Europe. An urban scaling approach to mapping the European economic transition," *PLoS ONE*, vol. 11, no. 8, pp. 1–8, 2016.

[8] L. M. Bettencourt, J. Lobo, and D. Strumsky, "Invention in the city: Increasing returns to patenting as a scaling function of metropolitan size," *Research Policy*, vol. 36, no. 1, pp. 107–120, 2007.

[9] F. F. L. Ribeiro, "An attempt to unify some population growth models from first principles," *Revista Brasileira de Ensino de Fisica*, vol. 39, no. 1, pp. 1–11, 2017.
